# Biochemical and molecular biomarkers: unraveling their role in gestational diabetes mellitus

**DOI:** 10.1186/s13098-023-00980-8

**Published:** 2023-01-11

**Authors:** Masoumeh Karami, Seyyed Hossein Mousavi, Mohammad Rafiee, Reza Heidari, Seyedeh Zahra Shahrokhi

**Affiliations:** 1 Department of Biochemistry, School of Medicine, AJA University of Medical Sciences, Tehran, Iran; 2 Department of Cardiology, School of Medicine, AJA University of Medical Sciences, Tehran, Iran; 3 Department of Medical Laboratory Sciences, School of Paramedical Sciences, Zanjan University of Medical Sciences, Zanjan, Iran; 4 Medical Biotechnology Research Center, AJA University of Medical Sciences, Tehran, Iran; 5 Research Center for Cancer Screening and Epidemiology, AJA University of Medical Sciences, Tehran, Iran

**Keywords:** β-cell dysfunction, Biomarker, Gestational diabetes mellitus, Insulin resistance, MicroRNA

## Abstract

Gestational diabetes mellitus (GDM) is the most prevalent metabolic disorder during pregnancy, causing short- and long-term complications for both mother and baby. GDM is a multifactorial disease, and it may be affected by interactions between genetic, epigenetic, and environmental factors. However, the exact etiology is poorly understood. Despite the high prevalence of GDM, there is still debate regarding the optimal time for screening, the diagnostic threshold to apply, and the best strategies for treatment. Identifying effective strategies for therapeutic purposes as well as accurate biomarkers for prognostic and diagnostic purposes will reduce the GDM incidence and improve its management. In recent years, new biochemical and molecular biomarkers such as microRNAs, single-nucleotide polymorphisms, and DNA methylation have received great interest in the diagnosis of GDM. In this review, we discuss current and future diagnostic approaches for the detection of GDM and evaluate lifestyle and pharmacological strategies for GDM prevention.

## Introduction

Gestational diabetes mellitus (GDM) is described as the type of glucose intolerance that occurs in the second and third trimesters of pregnancy, resulting in hyperglycemia of varying severity [1]. The global prevalence of GDM is increasing dramatically due to the global shift towards increasing rates of obesity, sedentary lifestyles, and advanced maternal age [2]. GDM affects between 2 and 38% of pregnancies [3], depending on the population investigated, as rates of GDM are higher in racial and ethnic minorities than in non-Hispanic white people [4]. Differences in the diagnostic criteria can also contribute to the variability in GDM prevalence, as its prevalence was 2.4 times greater when the International Association of Diabetes in Pregnancy Study Group (IADPSG) guideline were applied rather than the 1999 World Health Organization (WHO) guideline [5]. Interestingly, current evidence shows that the prevalence of GDM varies with the seasons, with greater rates in the summer and lower rates in the winter [6].

Untreated GDM can result in both short- and long-term complications for the mother and fetal health, including hypertension, preeclampsia, cesarean delivery, birth trauma, macrosomia, and neonatal hyperbilirubinemia [7, 8]. Children of women who have GDM are more prone to suffering from metabolic complications in later life [9]. Furthermore, it has been reported that women with GDM have a sevenfold increased risk of developing T2DM compared to women who have normal glucose tolerance [10], indicating that GDM prevalence could reflect the rate of type 2 diabetes mellitus (T2DM) in the general population [11]. Factors related to T2DM progression include the degree of abnormality on glucose tolerance tests, gestational age at GDM diagnosis, and insulin use during pregnancy [12]. However, a small percentage of women with the GDM who have pancreatic beta-cell autoantibodies may develop type 1 diabetes mellitus in the postpartum period [13].

Despite the fact that GDM is the most prevalent metabolic disease during pregnancy, there is considerable controversy about diagnostic methods, treatment options, and postpartum monitoring [14, 15]. Several studies have demonstrated that early diagnosis and treatment of GDM improves pregnancy outcomes, which highlights the importance of optimization strategies for its detection and treatment [16, 17]. Unfortunately, current diagnosis criteria, which are based on glucose homeostasis, are unable to predict or detect all cases of GDM [9]. For this reason, biomarkers for GDM should be selective and specific, easy to detect in circulation, non-invasive, and unaffected by normal metabolic and physiological changes [9]. As a result, the discovery of new biomarkers for the early detection of GDM could help clinicians better manage these patients and minimize the unfavorable pregnancy outcomes associated with GDM.

## Etiology and pathogenesis of GDM

Although the etiology of GDM is has not been fully established, there are several proposed mechanisms and risk factors that could help in order to understand the progression of this disease.

### Risk factor for GDM

Several risk factors for GDM have been identified through epidemiological studies, although the findings have been inconsistent [12, 18–20]. Obesity and a family history of diabetes are key risk factors for GDM. GDM risk is 2.14 times greater in obese women, 3.56 times higher in overweight women, and 8.56 times greater in extremely obese women compared to normal-weight pregnant women [20]. Advanced maternal age is a known risk factor for GDM. However, there is no agreement regarding an appropriate cut-off value [12]. The American Diabetes Association proposed using age 25 as a risk factor for GDM [21]. It has been shown that GDM was not prevalent in pregnant women under the age of 20, but it was found in 33.3% of pregnant women aged 20–29 and 58.3% of pregnant women aged 30–39 [19]. Interestingly, depression is currently recognized as a risk factor for GDM, with a 1.54-times increased odds of GDM [22].

### Healthy pregnancy vs. GDM pregnancy

During normal pregnancy, there are noteworthy changes in the metabolic profile of pregnant women, such as a decline in insulin sensitivity and elevating fatty acids and glucose levels to meet the physiological needs of the developing fetus [2]. As gestation progresses, it shown that a gradual rise in the levels of maretnal and placental hormones such as progesterone, estrogen, prolactin, placental growth hormone and human placental lactogen (hPL) accompanied by a state of insulin resistance [2]. Progesterone is implicated in insulin resistance by inhibiting the PI3-kinase pathway, and insulin receptor substrate 1 (IRS1) expression. Estradiol induced insulin resistance via membrane estrogen receptor (ER)-mediated activation of JNK and subsequent serine phosphorylation of IRS-1 [23]. Prolactin largely regulates its functions on β-cell through the JAK-2/signal transducer and activator of transcription (STAT)-5 pathway [24]. Human placental growth hormone (hPGH) may involved in insulin resistance by increasing the expression of the p85-regulatory unit of PI3K, resulting in a marked reduction in IRS-1-associated PI3K activity. Human placental lactogen (hPL) causes insulin resistance via decreasing [25] phosphorylation of IRS-1 [25]. It has been reported that first-and second-phase insulin responses to an intravenous glucose tolerance test were raised about threefold in late pregnancy compared to pre-pregnancy [26]. Finally, declined insulin sensitivity related to a normal pregnancy disappears within days of birth, indicating that these alterations are mediated by placental hormones [27].

**Fig. 1:**
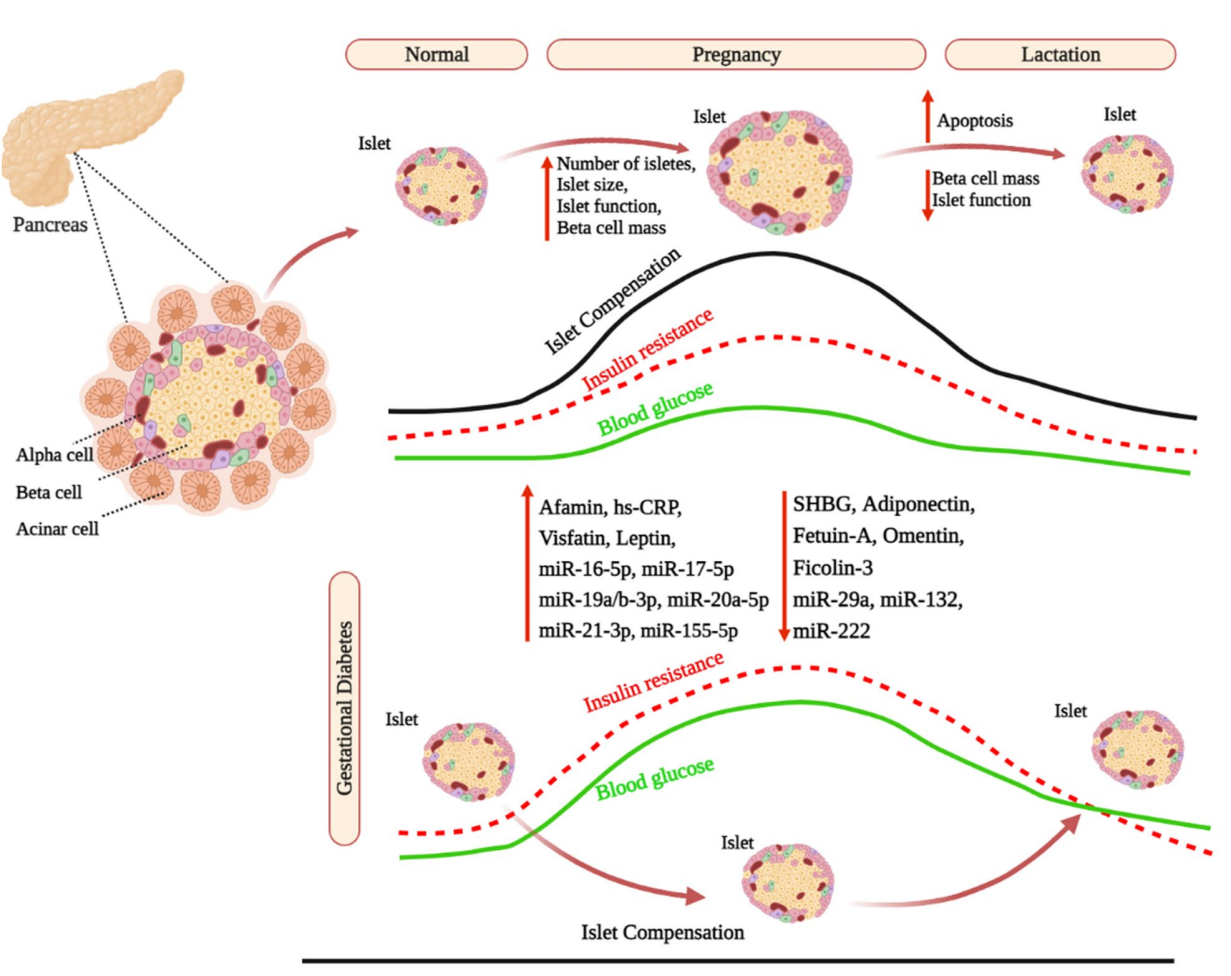
Alteration in insulin signaling pathway in normoglycemic and GDM pregnant women. Insulin signaling in skeletal muscle is mediated by tyrosine phosphorylation of the insulin receptor. This step activates downstream effectors like insulin receptor substrate 1 (IRS1) and phosphatidylinositol 3-kinase (PI3K), which allows glucose transporter type 4 (GLUT4) to translocate to the plasma membrane, resulting in enhanced glucose absorption. IRS1 levels in skeletal muscle are lower during pregnancy than in non-pregnant women. Furthermore, pregnancies with gestational diabetes mellitus (GDM) reduce tyrosine autophosphorylation of the insulin receptor β-subunit, resulting in a 25% decrease in glucose uptake when compared to pregnant with normoglycemic women

Usually, GDM appears between the 24th and 28th weeks of gestation and is associated with the failure of pancreatic β-cells to produce insulin, resulting in hyperglycemia to varying degrees [12]. When maternal insulin production is insufficient to compensate for the insulin-resistant condition, glucose intolerance develops, which may increase the chance of developing GDM (Fig. 1) [28]. Insulin resistance can develop as early as the second trimester, and by the third trimester and is enhanced by around 56% in patients due to impaired insulin signaling in skeletal muscle and adipose tissue [29]. Also, glucose consumption has been shown to decrease by 40–60%, depending on BMI [30]. In parallel with β-cell dysfunction, the insulin pathway appears to be altered.

**Fig. 2:**
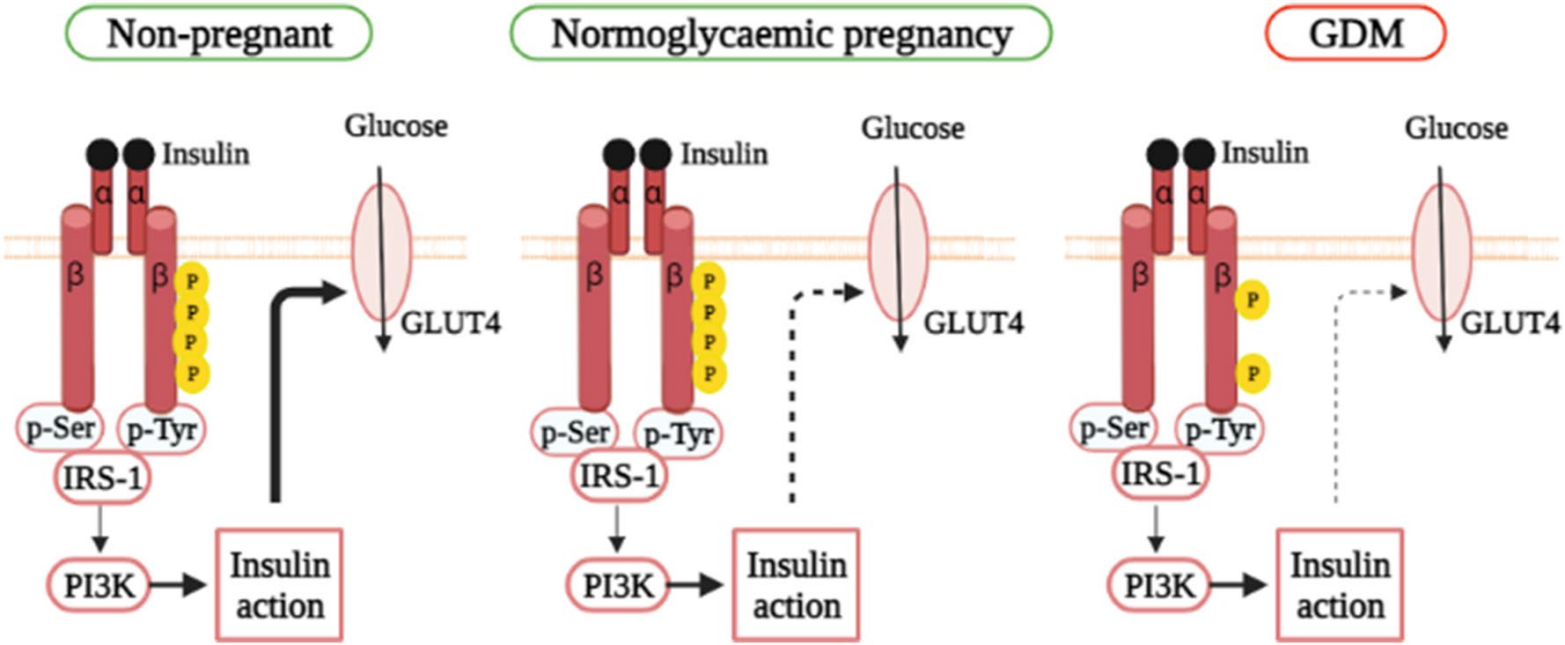
Changes in blood glucose levels, insulin resistance, islet compensation and miRNA profiles in GDM

In normal conditions, insulin binds to the insulin receptor on the cell membrane of peripheral tissues like skeletal muscle and causes glucose uptake by cells. This interaction causes autophosphorylation of the tyrosine kinase domain of the insulin receptor-β subunit (IR), resulting in the localization of glucose transporter type 4 (GLUT4) to the cell surface, allowing glucose absorption by the cell. IRS1 level, as one of the insulin signaling molecules, is decreased in pregnant compared to non-pregnant women [31]. Furthermore, a significant reduction in glucose uptake is observed in skeletal muscle of GDM women compared to those with normal glucose tolerance, due to lower autophosphorylation of IR (Fig. 2).

In addition to environmental factors such as obesity, recent studies have proposed that genetic and epigenetic factors play an important role in the pathogenesis of GDM. Evidence suggests that the link between genetics and GDM includes: (1) reappearance of GDM in at least 30% of previously diagnosed women; (2) genetic variations in genes involved in insulin production and resistance; and (3) heterogeneity in ethnic-group have been correlated to increased risk of GDM [32].

## Diagnosis of GDM

Early detection and accurate prediction of pregnant women with GDM is critical because it can reduce pregnancy-related problems in both the mother and the offspring. However, there is still no universal agreement on issues like screening time, diagnostic tests, and proper glycemic cut-offs for GDM [33]. Up to 2010, the two most commonly used criteria for GDM were by the World Health Organization (WHO) and the American Diabetes Association (ADA). The ADA recommended a 100-g OGTT at 14–18 weeks of gestation for high-risk women and 28–32 weeks for medium-risk women [1]. Currently, it has been recommended that GDM be diagnosed by the International Association of Diabetes and Pregnancy Study Group (IADPSG) criteria. The IADPSG criteria were established in response to the findings of the Hyperglycemia and Adverse Pregnancy Outcomes (HAPO) Study, a large international study involving 23,000 pregnant women [34]. The results of the HAPO Study showed that even when maternal glycemic was below the threshold for GDM diagnosis, there was a continued risk of adverse maternal and fetal outcomes, indicating that intervention criteria required to be modified. As a result, the IADPSG recommends that all pregnant women perform a fasting plasma glucose (FPG) test, glycosylated hemoglobin (HbA1c), or random plasma glucose at their first prenatal check (≥ 5.1 mmol/L indicates GDM) [34]. These glycemic thresholds are lower than other criteria. Today, the IADPSG criteria are the most commonly proposed guidelines, while some countries still use alternative criteria. Table 1 summarizes the most important screening and diagnosis guidelines for GDM. After considering the guidelines for GDM diagnosis, the identification of specific biomarkers in various phases of GDM pregnancy may be helpful for disease screening.

**Table 1:** Various guidelines for evaluating GDM based on glucose tolerance test

Criteria	Gestational age at screening (weeks)	Glucose load (g)	Glucose threshold (mmol/L)			
			Fasting	1 h	2 h	3 h
WHO, 1999	24–28	75	< 7.0	–	< 7.8	**–**
ADA, 2004	High risk: 14–18 Medium risk: 28–32	100	< 5.3	< 10.0	< 8.6	< 7.8
IADPSG, 2010	24–28	75	< 5.1	< 10	< 8.5	–
NICE, 2015	24–28	75	< 5.6	–	< 7.8	–
ACOG, 2018	24–28	100	< 5.3	< 10	< 8.6	< 7.8

*WHO* World Health Organization, *ADA* American Diabetes Association, *IADPSG* International Association of the Diabetes and Pregnancy Study Groups, *NICE* United Kingdom-based National Institute for Health and Care Excellence, *ACOG* American Congress of Obstetricians and Gynecologists

## Prospective diagnostic and predictive markers for GDM

During GDM, adipose and placenta tissue may release specific factors that contribute to inflammation and insulin resistance [9]. These biomarkers may be detected in maternal blood or urine and may be used to predict and/or detect GDM. In addition, the combination of biochemical and molecular biomarkers to create a predictive model may help in the early detection of GDM as well as the reduction of GDM-related complications.

### Biochemical markers

Several biochemical markers with their sensitivity and specificity shown in Table 2 (Part A). These biomarkers will be discussed as detailed in the following sections.

**Table 2:** Biochemical and molecular biomarkers for the prediction and detection of GDM

Part A						
Biochemical biomarker	Main source	Gestational age (weeks)	Sensitivity	Specificity	Change in GDM	References
SHBG	Liver, placenta	1st–13th	85.0	55.3	Lower	[48, 50]
Afamin	Liver, placenta	1st–12th	–	–	Higher	[52]
Ficolin-3	Liver, placenta	16th–18th	51.1	97.7	Lower	[39]
Fetuin-A	Liver, placenta	11–14th	58.6	76.2	Lower	[82]
hs-CRP	Liver, pancreas	4–6th/11–14th	89.0	55.3	Higher	[50]
Visfatin	Adipose, placenta	11–13th	–	–	Higher	[41]
Omentin-1	Adipose, placenta	12–15th	–	–	Lower	[45]
Adiponectin	Adipose, breast	6th–32nd	80.7	65.1	Lower	[39]
Leptin	Adipose, breast	14–20th/ 24–28th	81.2	64.2	Higher	[36]
1,5-anhydroglucitol	Liver	–	–	–	lower	[83]
Glycosylated fibronectin	Liver	–	–	–	Higher	[84]

Part B						
Molecular biomarker	Main source	Week of pregnancy	Sensitivity	Specificity	Change in GDM	References
miR-16-5p	Placenta	4–6th/16th	41.6	95.8	Higher	[85]
miR-17-5p	Placenta	4–6th/16th	21.4	95.4	Higher	[85]
miR-19a/b-3p	Placenta	16th	–	–	Higher	[9]
miR-20a-5p	Placenta	4–6th/16th	17.8	95.4	Higher	[85]
miR-21-3p	Placenta	7th–23rd/30–36th	52.6	89.3	Higher	[67]
miR-29a	Placenta	16th	–	–	Lower	[60]
miR-132	Placenta	16th	66.7	63.3	Lower	[60]
miR-155-5p	Placenta	16th	–	–	Higher	[66]
miR-222	Placenta	16th	–	–	Lower	[60]

Part C						
Urine biomarker	Main source	Week of pregnancy	Sensitivity	Specificity	Change in GDM	References
BHBA	Urine/Plasma	12th–28th	–	–	Higher	[55]
L-tryptophan	Urine/Plasma	12th–28th	–	–	Higher	[55]
Alanin	Urine/Plasma	12th–28th	–	–	Higher	[55]
Serotonin	Urine/Plasma	12th–28th	–	–	Higher	[55]

Part D						
Other biomarkers	Main source	Week of pregnancy	Sensitivity	Specificity	Change in GDM	References
IL-6	Adipose, lung	12–15th	–	–	Higher	[9]
TNFα	Macrophages (adipose, placenta)	24–28th	–	–	Higher	[9]
Mean platelet volume	Platelets	Second trimester	–	–	Higher	[86]
hsa_circRNA_0039480	Plasma-drived exosomal	First trimesters	54	93	Higher	[87]
hsa_circRNA_0054633	Placenta	Second and third trimesters	–	–	Higher	[88]

#### Adipose-related markers

Adipose tissue can secrete hormone-like substances called adipokines. A number of these compounds may be involved in the progression of diabetes [9]. Leptin, an adipocyte-derived hormone, is produced by adipocytes, the ovaries, and the placenta which influences glucose metabolism by inhibition of insulin action [35]. In both normal weight and obese women with GDM, leptin levels are considerably greater in the 2nd half of pregnancy than in non-GDM women [36]. In contrast, adiponectin, a protein produced by adipocytes, has anti-inflammatory and insulin-sensitizing properties [37]. Although adiponectin concentrations decrease during normal pregnancy, it has been reported that adiponectin concentrations in women with GDM are significantly lower compared to non-GDM women [37, 38]. Indeed, hypoadiponectinemia increased the risk of developing GDM by 4.6 times, and it was found to be inversely related to BMI, insulin resistance, and leptin levels [39]. As a result, GDM can be predicted by the plasma adiponectin/leptin ratio (0.33) in the 6th–14th week of pregnancy [40].

Other adipokines that may be used to diagnose GDM are visfatin, resistin, and omentin. Visfatin may trigger NF-κB signaling, which can contribute to insulin resistance [9]. Visfatin levels were elevated in the late first trimester [41], but were variably expressed in the third trimester of GDM [42]. Also, resistin, linked to elevated pro-inflammatory molecules, was reported to be decreased or unaffected by GDM [43]. Nevertheless, omentin-1 produced by non-fat cells in adipose tissue contributed to relaxation of vascular tone by increasing endothelial NO generation and decreasing hs-CRP and TNF signaling [44]. Omentin-1 levels were significantly decreased in the second trimester of GDM, similar to adiponectin [45]. It has been reported that women with GDM had lower fasting ghrelin levels than non-pregnant controls and pregnant women without GDM in the first and third trimesters of pregnancy [46].

#### Placenta-related markers

Placenta-related factors may involve in the progression of GDM. SHBG is a glycoprotein expressed in the placenta and involved in the transport of sex hormones and is suppressed by insulin [9]. SHBG levels have been found to be negatively associated with obesity and insulin resistance [47]. Interestingly, decreased plasma SHBG levels during the first trimester of pregnancy served as an accurate biomarker for GDM [48]. In a study conducted by Nanda et al., a decrease in SHBG levels occurred in parallel to an increase in adiponectin levels in GDM women during the 11–13th week, which was associated with a BMI > 30 kg/m^2^, and a family history of DM. Furthermore, women who were received insulin had even lowered SHBG levels [49]. More remarkably, a decrease in plasma fetuin-A concentrations and an increase in hs-CRP concentrations were also found in the late first trimester [50]. Fetuin-A was demonstrated to interact with the insulin receptor tyrosine kinase, resulting in the development of insulin resistance [51]. Furthermore, afamin, which is found in the liver and placenta and belongs to the albumin family, may serve as a biomarker for abnormal lipid and glucose metabolism in the first trimester [52]. In this regard, lower levels of ficolin-3, a lectin pathway activator found in the liver and placenta, and a higher ficolin-3/adiponectin ratio predicted GDM during the 16–18th week of pregnancy [37]. Finally, follistatin, known as a regulator of follicular-stimulant hormone, was reduced in the third trimester of GDM pregnancy [53].

#### Urine markers

Maternal urine may potentially contain prognostic and diagnostic markers related to GDM [9]. The evaluation of the urine samples from GDM women during the third trimester of pregnancy showed a significant elevation of 14 compounds associated with tryptophan metabolism and steroid hormone production during the third trimester of pregnancy [54]. Upregulation of these pathways during GDM may exacerbate insulin resistance and may be a response to oxidative damage and inflammation. Also, at the 12th–26th week of pregnancy, elevated levels of 3-hydroxybutanoic acid (BHBA), valine, and alanine were detected in both the urine and plasma of GDM women. Additionally, serotonin and associated metabolites, such as l-tryptophan, were also excreted more often in these patients [55].

### Molecular markers

There is growing evidence that genetic markers such as microRNAs, SNPs, and DNA methylation can be used as biomarkers for early detection of GDM [56]. Despite their potential, these molecular biomarkers present a number of challenges that must be overcome before they may be used in clinical settings.

#### MicroRNA

MiRNAs (miR) are small, approximately 22 nucleotides in length, highly conserved non-coding RNA molecules that act as potent regulators of biological function [57]. They have been associated with genes involved in insulin signaling and glucose and lipid metabolism [58].

MiRs could be secreted from the placenta into the maternal bloodstream as early as the 6th week of pregnancy and may play a role in placenta growth and insulin signaling [58]. These miRs are secreted either passively (associating with apoptotic bodies) or actively (as vesicles, exosomes, or lipoproteins) [9]. It has also been shown that miRs have an impact on proliferation, apoptosis, and angiogenesis in trophoblasts [59]. Therefore, their placental dysregulation was associated with metabolic conditions, including GDM. A significant reduction in serum levels of miR-29a, miR-132, and miR-222 was found in GDM women (n = 28) at the 16th week of pregnancy compared to controls (n = 53) [60]. MiR-29a was shown to be involved in glucose and fatty acid metabolism, whereas miR-222 has been related to insulin resistance and miR-132 has been associated with insulin secretion and glucose homeostasis [61, 62]. In contrast, Tagoma et al. observed unregulated plasma levels of miR-222 in women suffering GDM (n = 13) compared with controls (n = 9) [63]. These conflicting results could be explained by changes in the biological specimens (serum/plasma), maternal age, or other unknown variables. On the other hand, other miR such as miR-17-5p, miR-16-5p, and miR-20a-5p were increased in GDM during 1st–2nd trimester [64]. These miRs have been implicated in the regulation of inflammation and insulin resistance [65].

Similarly, unregulated plasma miR-21-3p levels were related to GDM during the 7th–23rd weeks of pregnancy. Notably, this miR has also been associated with insulin resistance and preeclampsia [66, 67]. Several miRs with their sensitivity and specificity are shown in Table 2 (Part B). These biomarkers can be used for early prediction of GDM in maternal plasma during pregnancy.

#### Single-nucleotide polymorphisms

Single-nucleotide polymorphisms (SNPs), the most abundant genetic variation in the DNA sequence, may have biologically functional effects and alter protein function [68]. Numerous SNPs in genes involved in insulin production and resistance, as well as glucose and lipid metabolism, have been associated with a higher risk of GDM [1, 16]. Mutations in adiponectin, glucokinase (GCK), glucokinase regulator (GCKR), insulin-like growth factor 2-binding protein (IGF2BP), insulin receptor substrate 1 (IRS-1), peroxisome proliferator-activated receptor gamma (PPARG2), among others, have been known [16]. Table 3 lists several SNPs that have been identified in numerous studies. Numerous studies found a positive or negative association, as well as no association, between various SNPs and GDM [69, 70]. These contradictory results could be explained by discrepancies in population and genotyping methods.

**Table 3:** Summary of studies evaluated important SNPs in GDM

Genes	Function	Variant	Detection method	Sample size (case/control)	Association	Risk for GDM	References
*ADIPOQ*	β-cell function	rs1501299	Realtime-PCR	204/207	No association	–	[89]
		rs266729	Realtime-PCR	204/207	G allele	Increased	[89]
		rs2241766	RFLP-PCR	65/70	G allele	Increased	[90]
*GCK*	β-cell function	rs1799884	Realtime-PCR	204/207	No association	–	[69]
			Realtime-PCR	278/179	T allele	Increased	[91]
		rs4607517	MassARRAY	533/407	No association	–	[92]
*GCKR*	β-cell function	rs780094	MassARRAY	267/855	C allele	Increased	[70]
			Realtime-PCR	204/207	No association	–	[69]
*IRS-1*	Insulin action	rs1801278	Realtime-PCR	278/179	No association	–	[91]
			RFLP	200/300	T allele	Increased	[93]
		rs7578326	MassARRAY	533/407	No association	–	[92]
			KASP	287/533	G allele	Decreased	[94]
*IGF2BP2*	β-cell function	rs4402960	Realtime-PCR	278/179	No association	–	[91]
			Realtime-PCR	725/1039	T allele	Increased	[95]
*PPARG2*	Insulin action	rs1801282	HRM	104/124	No association	–	[96]

*ADIPOQ* adiponectin, *GCK* glucokinase, *GCKR* glucokinase Regulator, *IGF2BP2* insulin-like growth factor 2 messenger RNA (mRNA)-binding protein 2, *IRS1* insulin receptor substrate 1, *PPARG2* peroxisome proliferator-activated receptor gamma 2

#### Epigenetic modifications

Epigenetic modifications are defined as variations in the molecular structure of DNA that result in alterations in gene expression [71]. Epigenetic mechanisms have an impact on several cellular activities linked with pathophysiological conditions [16]. Particularly, epigenetic modifications affect gene expression involved in beta-cell morphology, function, and proliferation, which indicates that epigenetic changes may impair insulin secretion and sensitivity [56].

DNA methylation, the most commonly studied epigenetic change, occurs when a methyl group is added to a cytosine residue in a cytosine–phosphate–guanine (CpG) sequence [71]. Several studies have indicated that DNA methylation pattern in the placenta and cord blood of women with GDM differs from that of women who are pregnant with normoglycemic pregnancies [72, 73]. A study by Kang et al. reported that IL-10 methylation decreased in GDM whereas IL-10 serum levels increase at the end of pregnancy [74]. DNA methylation might also be involved in the pathogenesis of childhood cardiometabolic traits via vascular adhesion molecule 1 (VCAM-1) [75]. It has been shown that VCAM-1 levels were elevated in offspring exposed to GDM in utero compared to non-exposed offspring, which may be related to elevated methylation of PYGO1 and CLN8 genes [75, 76]. Furthermore, DNA methylation near the leptin gene promoter affects the leptin levels in cord blood samples, and the results show that hypomethylation is associated with high leptin levels in the offspring [75]. DNA methylation may be a useful diagnostic and prognostic marker, however, the majority of studies reported in the context of GDM have a small sample size, and use various methodologies to detect DNA methylation.

## Treatment of GDM

The treatment of GDM can improve abnormal glucose tolerance and reduce the incidence of GDM consequence including preeclampsia and macrosomia.

**Fig. 3:**
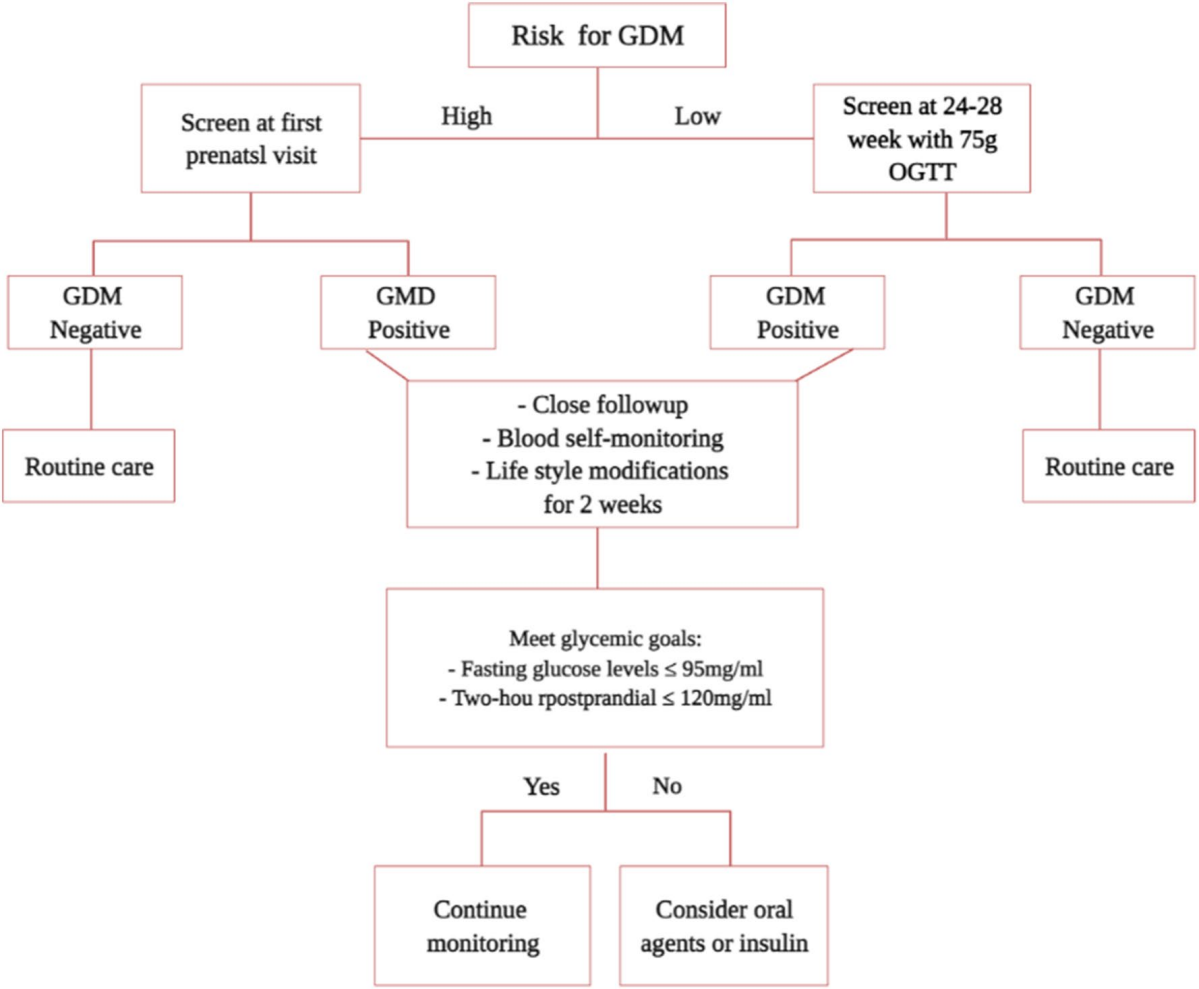
Proposed algorithm for management of patients in gestational diabetes

The first line of treatment for GDM is lifestyle interventions such as diet modifications and physical activity. Women with GDM should obtain specific dietary counseling, which generally recommends limiting carbohydrate intake to 33 to 40% of calories. Most women with GDM can achieve euglycemia with diet alone, with 15% to 30% of them needing insulin [77]. There are conflicting results regarding the effects of exercise on women with GDM [77, 78]. It has been demonstrated that aerobic exercise enhances glycemic control in diabetic patients, and exercising for 30 min most days of the week is an acceptable objective for the majority of individuals with GDM [78]. It is also important to monitor maternal weight gain in order to reduce the risk of fetal macrosomia. Women with obesity, GDM, and a gestational weight gain of more than 40 lb (18.1 kg) have a 40% risk of having fetal macrosomia [79]. When lifestyle interventions are ineffective for reducing blood glucose levels, pharmaceutical therapy is recommended (Fig. 3) [80]. Insulin and oral agents like metformin, and glyburide are used to treat the majority of GDM patients.

Metformin dosage depends on glycemic levels and begins at 500 mg every night or twice daily. During pregnancy, the maximum daily dose is 2500–3000 mg, which is more than the non-pregnant state [2]. Recently, the mata-analysis study has shown that metformin may have potential benefits for pregnant women and newborns with no obvious adverse effects. In spite of the fact that metformin can pass through the placenta and into the fetus, but the teratogenic effects of metformin have not been confirmed [81]. However, even more, studies are needed to provide evidence for the future use of metformin.

In order to the dosage of glyburide, it is gradually increased from 2.5 mg once every 12 h to a maximum of 10 mg twice daily, depending on glycemic control [45]. When oral agents fail to provide adequate glucose control, the patient should be re-addressed with the insulin option. Insulin is a relatively big molecule that does not pass the placenta during pregnancy. The dosage, type, and timing of insulin administration are customized for each patient and determined by a variety of factors, including the patient's weight, maternal age, and time of day [80]. As a result, since oral agents can have potentially side effects on pregnant women and fetuses, insulin is the recommended treatment, however, costs and patient experiences limit its use in clinical practice.

Treatment for GDM during pregnancy is controversial be the lack of data from large randomized clinical trials. There is an urgent need for well-designed study that can inform decisions on the best practice regarding GDM screening and diagnosis.

## Conclusion

The management of women with GDM is an increasingly common global health problem, since none of the existing biomarkers show high specificity for GDM diagnosis. Therefore, there is a need to detect accurate biomarkers of GDM to improve diagnostic power and facilitate clinical management. The biochemical and molecular markers, SNPs, and DNA methylation is potential biomarkers for early detection of GDM, however, they face several challenges that need to be addressed before be applied clinically. Future, studies are needed to calculate their decision threshold and to evaluate their clinical usefulness, as well as analytical sensitivity and specificity of the methods used.

## Data Availability

Not applicable.

## References

[1] Chiefari E, Arcidiacono B, Foti D, Brunetti A (2017). Gestational diabetes mellitus: an updated overview. J Endocrinol Investig.

[2] Johns EC, Denison FC, Norman JE, Reynolds RM (2018). Gestational diabetes mellitus: mechanisms, treatment, and complications. Trends Endocrinol Metab.

[3] Alesi S, Ghelani D, Rassie K, Mousa A (2021). Metabolomic biomarkers in gestational diabetes mellitus: a review of the evidence. Int J Mol Sci.

[4] Lawrence JM, Contreras R, Chen W, Sacks DA (2008). Trends in the prevalence of preexisting diabetes and gestational diabetes mellitus among a racially/ethnically diverse population of pregnant women, 1999–2005. Diabetes Care.

[5] Jenum AK, Mrøkrid K, Sletner L, Vange S, Torper JL, Nakstad B (2012). Impact of ethnicity on gestational diabetes identified with the WHO and the modified International Association of Diabetes and Pregnancy Study Groups criteria: a population-based cohort study. Eur J Endocrinol.

[6] Moses RG, Wong VCK, Lambert K, Morris GJ, Gil FS (2016). Seasonal changes in the prevalence of gestational diabetes mellitus. Diabetes Care.

[7] Burlina S, Dalfrà MG, Lapolla A (2019). Short- and long-term consequences for offspring exposed to maternal diabetes: a review. J Matern Neonatal Med.

[8] Lowe WL, Scholtens DM, Lowe LP, Kuang A, Nodzenski M, Talbot O (2018). Association of gestational diabetes with maternal disorders of glucose metabolism and childhood adiposity. JAMA J Am Med Assoc.

[9] Lorenzo-Almorós A, Hang T, Peiró C, Soriano-Guillén L, Egido J, Tuñón J (2019). Predictive and diagnostic biomarkers for gestational diabetes and its associated metabolic and cardiovascular diseases. Cardiovasc Diabetol.

[10] Damm P, Houshmand-Oeregaard A, Kelstrup L, Lauenborg J, Mathiesen ER, Clausen TD (2016). Gestational diabetes mellitus and long-term consequences for mother and offspring: a view from Denmark. Diabetologia.

[11] Zhu Y, Zhang C (2016). Prevalence of gestational diabetes and risk of progression to type 2 diabetes: a global perspective. Curr Diab Rep.

[12] Choudhury AA, Devi Rajeswari V (2021). Gestational diabetes mellitus—a metabolic and reproductive disorder. Biomed Pharmacother.

[13] Ikeoka T, Sako A, Kuriya G, Yamashita H, Yasuhi I, Horie I (2018). Type 1 diabetes mellitus diagnosed during follow-up of gestational diabetes mellitus in the early postpartum period. Intern Med.

[14] Dalfrà MG, Burlina S, Del Vescovo GG, Lapolla A (2020). Genetics and epigenetics: new insight on gestational diabetes mellitus. Front Endocrinol.

[15] Liu X, Wu N, Al-Mureish A (2021). A review on research progress in the application of glycosylated hemoglobin and glycated albumin in the screening and monitoring of gestational diabetes. Int J Gen Med.

[16] Dias S, Pheiffer C, Abrahams Y, Rheeder P, Adam S (2018). Molecular biomarkers for gestational diabetes mellitus. Int J Mol Sci.

[17] Addison B, Belalcazar LM (2014). Introduction of IADPSG criteria for the screening and diagnosis of gestational diabetes mellitus results in improved pregnancy outcomes at a lower cost in a large cohort of pregnant women: the St. Carlos gestational diabetes study. Diabetes Care.

[18] Li G, Wei T, Ni W, Zhang A, Zhang J, Xing Y (2020). Incidence and risk factors of gestational diabetes mellitus: a prospective cohort study in Qingdao, China. Front Endocrinol.

[19] Ngala RA, Fondjo LA, Gmagna P, Ghartey FN, Awe MA (2017). Placental peptides metabolism and maternal factors as predictors of risk of gestational diabetes in pregnant women. A case-control study. PLoS ONE.

[20] Chu SY, Callaghan WM, Kim SY, Schmid CH, Lau J, England LJ (2007). Maternal obesity and risk of gestational diabetes mellitus. Diabetes Care.

[21] Care ADA-D (2017). Classification and diagnosis of diabetes. Diabetes Care.

[22] Larrabure-Torrealva GT, Martinez S, Luque-Fernandez MA, Sanchez SE, Mascaro PA, Ingar H (2018). Prevalence and risk factors of gestational diabetes mellitus: findings from a universal screening feasibility program in Lima, Peru. BMC Pregnancy Childbirth.

[23] Wada T, Hori S, Sugiyama M, Fujisawa E, Nakano T, Tsuneki H (2010). Progesterone inhibits glucose uptake by affecting diverse steps of insulin signaling in 3T3-L1 adipocytes. Am J Physiol Endocrinol Metab.

[24] Gorvin CM (2015). The prolactin receptor: diverse and emerging roles in pathophysiology. J Clin Transl Endocrinol.

[25] Barbour LA, Shao J, Qiao L, Leitner W, Anderson M, Friedman JE (2004). Human placental growth hormone increases expression of the P85 regulatory unit of phosphatidylinositol 3-kinase and triggers severe insulin resistance in skeletal muscle. Endocrinology.

[26] Catalano PM, Tyzbir ED, Wolfe RR, Calles J, Roman NM, Amini SB (1993). Carbohydrate metabolism during pregnancy in control subjects and women with gestational diabetes. Am J Physiol Endocrinol Metab.

[27] Plows JF, Stanley JL, Baker PN, Reynolds CM, Vickers MH (2018). The pathophysiology of gestational diabetes mellitus. Int J Mol Sci.

[28] Kramer CK, Swaminathan B, Hanley AJ, Connelly PW, Sermer M, Zinman B (2014). Each degree of glucose intolerance in pregnancy predicts distinct trajectories of β-cell function, insulin sensitivity, and glycemia in the first 3 years postpartum. Diabetes Care.

[29] Colomiere M, Permezel M, Riley C, Desoye G, Lappas M (2009). Defective insulin signaling in placenta from pregnancies complicated by gestational diabetes mellitus. Eur J Endocrinol.

[30] Sivan E, Chen X, Homko CJ, Reece EA, Boden G (1997). Longitudinal study of carbohydrate metabolism in healthy obese pregnant women. Diabetes Care.

[31] Nguyen-Ngo C, Jayabalan N, Salomon C, Lappas M (2019). Molecular pathways disrupted by gestational diabetes mellitus. J Mol Endocrinol.

[32] Yuen L (2015). Gestational diabetes mellitus: challenges for different ethnic groups. World J Diabetes.

[33] Agarwal MM (2015). Gestational diabetes mellitus: an update on the current international diagnostic criteria. World J Diabetes.

[34] Basri NI, Mahdy ZA, Ahmad S (2018). The World Health Organization (WHO) versus the International Association of Diabetes and Pregnancy Study Group (IADPSG) diagnostic criteria of gestational diabetes mellitus (GDM) and their associated maternal and neonatal outcomes. Horm Mol Biol Clin Investig.

[35] Katsiki N, Mikhailidis DP, Banach M (2018). Leptin, cardiovascular diseases and type 2 diabetes mellitus review. Acta Pharmacol Sin.

[36] Boyadzhieva M, Atanasova I, Zacharieva S, Kedikova S (2013). Adipocytokines during pregnancy and postpartum in women with gestational diabetes and healthy controls. J Endocrinol Investig.

[37] Yuan XS, Shi H, Wang HY, Yu B, Jiang J (2018). Ficolin-3/adiponectin ratio for the prediction of gestational diabetes mellitus in pregnant women. J Diabetes Investig.

[38] Bozkurt L, Göbl CS, Baumgartner-Parzer S (2018). Adiponectin and leptin at early pregnancy: association to actual glucose disposal and risk for GDM—a prospective cohort study. Int J Endocrinol.

[39] Cseh K, Baranyi É, Melczer Z, Kaszás E, Palik É, Winkler G (2004). Plasma adiponectin and pregnancy-induced insulin resistance. Diabetes Care.

[40] Thagaard IN, Krebs L, Holm JC, Lange T, Larsen T, Christiansen M (2017). Adiponectin and leptin as first trimester markers for gestational diabetes mellitus: a cohort study. Clin Chem Lab Med.

[41] Ferreira AFA, Rezende JC, Vaikousi E, Akolekar R, Nicolaides KH (2011). Maternal serum visfatin at 11–13 weeks of gestation in gestational diabetes mellitus. Clin Chem.

[42] Rezvan N, Hosseinzadeh-Attar MJ, Masoudkabir F, Moini A, Janani L, Mazaherioun M (2012). Serum visfatin concentrations in gestational diabetes mellitus and normal pregnancy. Arch Gynecol Obstet.

[43] Megia A, Vendrell J, Gutierrez C, Sabaté M, Broch M, Fernández-Real JM (2008). Insulin sensitivity and resistin levels in gestational diabetes mellitus and after parturition. Eur J Endocrinol.

[44] Yang RZ, Lee MJ, Hu H, Pray J, Wu HB, Hansen BC (2006). Identification of omentin as a novel depot-specific adipokine in human adipose tissue: possible role in modulating insulin action. Am J Physiol Endocrinol Metab.

[45] Abell SK, Shorakae S, Harrison CL, Hiam D, Moreno-Asso A, Stepto NK (2017). The association between dysregulated adipocytokines in early pregnancy and development of gestational diabetes. Diabetes Metab Res Rev.

[46] Stranak Z, Krofta L, Haak LA, Vojtěch J, Hašlík L, Feyereisl J (2016). Prenatal parameters to estimate outcome and respiratory morbidity in fetuses with isolated left-sided congenital diaphragmatic hernia. Eur J Obstet Gynecol Reprod Biol.

[47] Ding EL, Song Y, Malik VS, Liu S (2006). Sex differences of endogenous sex hormones and risk of type 2 diabetes: a systematic review and meta-analysis. J Am Med Assoc.

[48] Zhang T, Du T, Li W, Yang S, Liang W (2018). Sex hormone-binding globulin levels during the first trimester may predict gestational diabetes mellitus development. Biomark Med.

[49] Nanda S, Savvidou M, Syngelaki A, Akolekar R, Nicolaides KH (2011). Prediction of gestational diabetes mellitus by maternal factors and biomarkers at 11 to 13 weeks. Prenat Diagn.

[50] Kansu-Celik H, Ozgu-Erdinc AS, Kisa B, Findik RB, Yilmaz C, Tasci Y (2019). Prediction of gestational diabetes mellitus in the first trimester: Comparison of maternal fetuin-A, N-terminal proatrial natriuretic peptide, high-sensitivity C-reactive protein, and fasting glucose levels. Arch Endocrinol Metab.

[51] Farhan S, Handisurya A, Todoric J (2012). Fetuin-A characteristics during and after pregnancy: Result from a case control pilot study. Int J Endocrinol.

[52] Köninger A, Mathan A, Mach P, Frank M, Schmidt B, Schleussner E (2018). Is Afamin a novel biomarker for gestational diabetes mellitus? A pilot study. Reprod Biol Endocrinol.

[53] Näf S, Escote X, Ballesteros M, Yañez RE, Simón-Muela I, Gil P (2014). Serum activin A and follistatin levels in gestational diabetes and the association of the activin A-follistatin system with anthropometric parameters in offspring. PLoS ONE.

[54] López-Hernández Y, Van Oostdam ASH, Toro-Ortiz JC, López JA, Salgado-Bustamante M, Murgu M (2019). Urinary metabolites altered during the third trimester in pregnancies complicated by gestational diabetes mellitus: relationship with potential upcoming metabolic disorders. Int J Mol Sci.

[55] Leitner M, Fragner L, Danner S, Holeschofsky N, Leitner K, Tischler S (2017). Combined metabolomic analysis of plasma and urine reveals AHBA, tryptophan and serotonin metabolism as potential risk factors in Gestational Diabetes Mellitus (GDM). Front Mol Biosci.

[56] Yahaya TO, Salisu T, Abdulrahman YB, Umar AK (2020). Update on the genetic and epigenetic etiology of gestational diabetes mellitus: a review. Egypt J Med Hum Genet.

[57] O’Brien J, Hayder H, Zayed Y, Peng C (2018). Overview of microRNA biogenesis, mechanisms of actions, and circulation. Front Endocrinol.

[58] Poirier C, Desgagné V, Guérin R, Bouchard L (2017). MicroRNAs in pregnancy and gestational diabetes mellitus: emerging role in maternal metabolic regulation. Curr Diab Rep.

[59] Morales-Prieto DM, Ospina-Prieto S, Schmidt A, Chaiwangyen W, Markert UR (2014). Elsevier trophoblast research award lecture: Origin, evolution and future of placenta miRNAs. Placenta.

[60] Zhao C, Dong J, Jiang T, Shi Z, Yu B, Zhu Y (2011). Early second-trimester serum miRNA profiling predicts gestational diabetes mellitus. PLoS ONE.

[61] He A, Zhu L, Gupta N, Chang Y, Fang F (2007). Overexpression of micro ribonucleic acid 29, highly up-regulated in diabetic rats, leads to insulin resistance in 3T3-L1 adipocytes. Mol Endocrinol.

[62] Shi Z, Zhao C, Guo X, Ding H, Cui Y, Shen R (2014). Differential expression of micrornas in omental adipose tissue from gestational diabetes mellitus subjects reveals mir-222 as a regulator of erα expression in estrogen-induced insulin resistance. Endocrinology.

[63] Tagoma A, Alnek K, Kirss A, Uibo R, Haller-Kikkatalo K (2018). MicroRNA profiling of second trimester maternal plasma shows upregulation of miR-195-5p in patients with gestational diabetes. Gene.

[64] Zhu Y, Tian F, Li H, Zhou Y, Lu J, Ge Q (2015). Profiling maternal plasma microRNA expression in early pregnancy to predict gestational diabetes mellitus. Int J Gynecol Obstet.

[65] Fernandes T, Magalhães FC, Roque FR, Phillips MI, Oliveira EM (2012). Exercise training prevents the microvascular rarefaction in hypertension balancing angiogenic and apoptotic factors: role of microRNAs-16, -21, and -126. Hypertension.

[66] Wander PL, Boyko EJ, Hevner K, Parikh VJ, Tadesse MG, Sorensen TK (2017). Circulating early- and mid-pregnancy microRNAs and risk of gestational diabetes. Diabetes Res Clin Pract.

[67] Hocaoglu M, Demirer S, Senturk H, Turgut A, Komurcu-Bayrak E (2019). Differential expression of candidate circulating microRNAs in maternal blood leukocytes of the patients with preeclampsia and gestational diabetes mellitus. Pregnancy Hypertens.

[68] Consortium T international H (2005). A second generation human haplotype map of over 3.1 million SNPs. Nature.

[69] Tarnowski M, Malinowski D, Pawlak K, Dziedziejko V, Safranow K, Pawlik A (2017). GCK, GCKR, FADS1, DGKB/TMEM195 and CDKAL1 gene polymorphisms in women with gestational diabetes. Can J Diabetes.

[70] Jamalpour S, Zain SM, Mosavat M, Mohamed Z, Omar SZ (2018). A case-control study and meta-analysis confirm glucokinase regulatory gene rs780094 is a risk factor for gestational diabetes mellitus. Gene.

[71] Lim DHK, Maher ER (2010). DNA methylation: a form of epigenetic control of gene expression. Obstet Gynaecol.

[72] Finer S, Mathews C, Lowe R, Smart M, Hillman S, Foo L (2014). Maternal gestational diabetes is associated with genome-wide DNA methylation variation in placenta and cord blood of exposed offspring. Hum Mol Genet.

[73] Reichetzeder C, Dwi Putra SE, Pfab T, Slowinski T, Neuber C, Kleuser B (2016). Increased global placental DNA methylation levels are associated with gestational diabetes. Clin Epigenet.

[74] Kang J, Lee CN, Li HY, Hsu KH, Wang SH, Lin SY (2018). Association of interleukin-10 methylation levels with gestational diabetes in a Taiwanese population. Front Genet.

[75] Elliott HR, Sharp GC, Relton CL, Lawlor DA (2019). Epigenetics and gestational diabetes: a review of epigenetic epidemiology studies and their use to explore epigenetic mediation and improve prediction. Diabetologia.

[76] West NA, Kechris K, Dabelea D (2013). Exposure to maternal diabetes in utero and DNA methylation patterns in the offspring. Immunometabolism.

[77] Dolatkhah N, Hajifaraji M, Shakouri SK (2018). Nutrition therapy in managing pregnant women with gestational diabetes mellitus: a literature review. J Fam Reprod Heal.

[78] Padayachee C (2015). Exercise guidelines for gestational diabetes mellitus. World J Diabetes.

[79] Black MH, Sacks DA, Xiang AH, Lawrence JM (2013). The relative contribution of prepregnancy overweight and obesity, gestational weight gain, and IADPSG-defined gestational diabetes mellitus to fetal overgrowth. Diabetes Care.

[80] Lende M, Rijhsinghani A (2020). Gestational diabetes: overview with emphasis on medical management. Int J Environ Res Public Health.

[81] Bao LX, Shi WT, Han YX (2021). Metformin versus insulin for gestational diabetes: a systematic review and meta-analysis. J Matern Neonatal Med.

[82] Salmi AA, Zaki NMN, Zakaria R, Aliza AGN, Rasool AHG (2012). Arteriensteifheit, entzändliche und proatherogene marker des gestationsdiabetes. Vasa J Vasc Dis.

[83] Corcoran SM, Achamallah N, Loughlin JO, Stafford P, Dicker P, Malone FD (2018). First trimester serum biomarkers to predict gestational diabetes in a high-risk cohort: Striving for clinically useful thresholds. Eur J Obstet Gynecol Reprod Biol.

[84] Rasanen JP, Snyder CK, Rao PV (2015). Glycosylated fibronectin as a first-trimester biomarker for prediction of gestational diabetes. Diabetes Technol Ther.

[85] Cao YL, Jia YJ, Xing BH, Shi DD, Dong XJ (2017). Plasma microRNA-16-5p, -17-5p and -20a-5p: novel diagnostic biomarkers for gestational diabetes mellitus. J Obstet Gynaecol Res Blackwell Publishing.

[86] Zhou Z, Chen H, Sun M, Ju H (2018). Mean platelet volume and gestational diabetes mellitus: a systematic review and meta-analysis. J Diabetes Res.

[87] Jiang B, Zhang J, Sun X, Yang C, Cheng G, Xu M (2022). Circulating exosomal hsa_circRNA_0039480 is highly expressed in gestational diabetes mellitus and may be served as a biomarker for early diagnosis of GDM. J Transl Med.

[88] Wu H, Wu S, Zhu Y, Ye M, Shen J, Liu Y (2019). Hsa-circRNA-0054633 is highly expressed in gestational diabetes mellitus and closely related to glycosylation index. Clin Epigenet.

[89] Pawlik A, Teler J, Maciejewska A, Sawczuk M, Safranow K, Dziedziejko V (2017). Adiponectin and leptin gene polymorphisms in women with gestational diabetes mellitus. J Assist Reprod Genet.

[90] Takhshid MA, Haem Z, Aboualizadeh F (2015). The association of circulating adiponectin and + 45 T/G polymorphism of adiponectin gene with gestational diabetes mellitus in Iranian population. J Diabetes Metab Disord.

[91] Popova PV, Klyushina AA, Vasilyeva LB, Tkachuk AS, Bolotko YA, Gerasimov AS (2017). Effect of gene-lifestyle interaction on gestational diabetes risk. Oncotarget.

[92] Huopio H, Cederberg H, Vangipurapu J, Hakkarainen H, Paakkonen M, Kuulasmaa T (2013). Association of risk variants for type 2 diabetes and hyperglycemia with gestational diabetes. Eur J Endocrinol.

[93] Alharbi KK, Khan IA, Abotalib Z, Al-Hakeem MM (2014). Insulin receptor substrate-1 (IRS-1) Gly927Arg: Correlation with gestational diabetes mellitus in Saudi women. Biomed Res Int.

[94] Rosta K, Al-Aissa Z, Hadarits O, Harreiter J, Nádasdi Á, Kelemen F (2017). Association study with 77 SNPs confirms the robust role for the rs10830963/G of MTNR1B variant and identifies two novel associations in gestational diabetes mellitus development. PLoS ONE.

[95] Wang Y, Wang O, Li W (2015). Variants in vitamin D binding protein gene are associated with gestational diabetes mellitus. Medicine.

[96] Franzago M, Fraticelli F, Marchetti D, Celentano C, Liberati M, Stuppia L (2018). Nutrigenetic variants and cardio-metabolic risk in women with or without gestational diabetes. Diabetes Res Clin Pract.

